# The National Relief Fund

**Published:** 1920-06-12

**Authors:** 


### THE NATIONAL RELIEF FUND.

The Report on the Administration of the National Relief Fund, which details the transactions of the Fund up to June 30, 1919, shows that the total contributions up to that date amounted to £5,870,797. In addition, the sum of £566,736 has been received in respect of interest earned on the balance of the Fund temporarily invested; and out of the amounts collected on behalf of the Queen's "Work for Women" Fund, the sum of £128,000 has been paid over to the National Relief Fund for the purpose of financing schemes for work for women. The aggregate receipts, therefore, up to June 30 amounted to £6,565,533. The total grants made for naval and military relief amounted to £3,223,267, and for civil relief, £976,597. There remained in hand on June 30 the sum of £2,495,334. The total expenses of administration have been £12,389, and towards this the Prince of Wales has personally given £3,498 to pay the wages of the clerical staff.

### The Future of the Fund.

The abnormally favourable conditions of employment which obtained during the latter part of the war considerably lightened the claims upon the Fund, but the Committee anticipated that the balance would be no more than sufficient for the relief of civil distress during the period of transition from war to peace industries. In fact, it was to avoid such unavoidable distress that the Government instituted the system of out-of-work donations, and thus postponed the expected call upon the resources of the Fund.

A change in the outlook having now come about, a sub-committee has been appointed to consider the future of the Fund and to review the various applications which have been made in the light of the new position. This sub-committee has recommended a number of grants, but the Committee of the Fund has not yet decided upon the final allocation of the balance in hand.

### Present Methods of Relief.

Recognising that the Government allowances to demobilised sailors and soldiers do no more than cover the bare cost of maintenance while they are becoming settled in peace occupations, the Fund has granted £100,000 to the Soldiers' and Sailors' Help Society, and £50,000 to the Soldiers' and Sailors' Families Association. In both cases these grants were made on the understanding that they should be accepted as final and in full discharge of any claims which these organisations might have against the Fund in respect of assistance to demobilised men. On similar grounds £10,000 has been given to the Imperial Association for Assisting Naval and Military Officers and the Disabled Officers' Fund, and some provision has been made towards a scheme for providing flats for disabled officers and officers' widows; £10,000 has been given to the Scottish Hostel for Blinded Sailors and Soldiers at Edinburgh.

In respect of civil distress, the attention of the Committee has been directed to the relief of lodging-house keepers and others on the East Coast. It is recognised that much money was made during the holiday season of 1919, but there was a great deal of lee-way to make up, and a sum of £10,000 was given to the East Coast Conference for the purpose. A smaller grant was made to air-raid victims.

### Education of Young War-workers.

As the Ministry of Reconstruction has said, "the boys and girls between fourteen and eighteen who have been 'abnormally employed' during the war have very special claims to consideration; but unless a vigilant public opinion is directed towards their needs, they are only too likely to be overlooked amid the claims of more vocal and better-organised workers." The Committee of the Fund has been approached by the Board of Education on behalf of this class, whose position is a serious one and of considerable economic importance. Discipline has been lost, physique has been prematurely worn out by long hours of work, and, above all, a considerable proportion of children have been obliged to forego, as a direct result of the war, their normal chances of receiving proper training for skilled trades. Should they drift into blind-alley occupations, they will represent a permanent loss to the nation.

Local education authorities in the districts concerned (chieny munition centres) are willing to provide training facilities and, the Treasury having also made a grant, the Relief Fund has set aside £100,000 to cover what is required in the United Kingdom. In England and Wales the Fund will be administerd by a Central Committee, and in Scotland by the Scottish Education Department.

### Sundry Relief.

The Fund has also made provision for distress amongst British refugees and repatriated civilian prisoners, re-equipment of distressed fishermen, to increase pensions of war cases of the Royal United Kingdom Beneficent Association, and for unemployment in Scotland and Ireland.

Roughly, the grants made by the Fund, as detailed above, amount to £300,000. Taking into consideration the accrued interest on balances since June 30, 1919, there should still be in hand well over two million pounds. In the light of its resources, therefore, the Fund cannot be accused of having erred on the side of prodigality.

